# Racial/ethnic, age and sex disparities in leukemia survival among adults in the United States during 1973-2014 period

**DOI:** 10.1371/journal.pone.0220864

**Published:** 2019-08-19

**Authors:** Ovie Utuama, Fahad Mukhtar, Yen Thi-Hai Pham, Bashir Dabo, Priyashi Manani, Jenna Moser, Abimbola Michael-Asalu, Chi TD Tran, Linh C. Le, Thanh V. Le, Khanh Truong Vu, Jong Y. Park, Paolo Boffetta, Wei Zheng, Xiao-Ou Shu, Hung N. Luu

**Affiliations:** 1 Department of Epidemiology and Biostatistics, College of Public Health, University of South Florida, Tampa, FL, United States of America; 2 Department of Rehabilitation, Vinmec Times City Hospital, Vinmec Healthcare System, Hanoi, Vietnam; 3 College of Arts and Sciences, University of South Florida, Tampa, FL, United States of America; 4 Vietnam Colorectal Cancer and Research Program, Vinmec Healthcare System, Hanoi, Vietnam; 5 College of Health Sciences, VinUniversity (VinUni), Hanoi, Vietnam; 6 Department of Hepatobiliary and Pancreatic Surgery, 108 Hospital, Hanoi, Vietnam; 7 Department of Gastroenterology, Bach Mai Hospital, Hanoi, Vietnam; 8 Department of Cancer Epidemiology, H. Lee Moffitt Cancer Center and Research Institute, Tampa, FL, United States of America; 9 Tisch Cancer Institute, Icahn School of Medicine, Mount Sinai School of Medicine, New York, NY, United States of America; 10 Division of Epidemiology, Department of Medicine, Vanderbilt Epidemiology Center, Vanderbilt-Ingram Cancer Center, Vanderbilt University School of Medicine, Nashville, TN, United States of America; 11 Department of Epidemiology, University of Pittsburgh Graduate School of Public Health, Pittsburgh, PA, United States of America; 12 Currently at the Division of Cancer Control and Population Sciences, University of Pittsburgh Cancer Institute, Pittsburgh, PA, United States of America; National Health Research Institutes, TAIWAN

## Abstract

There has been marked improvement in leukemia survival, particularly among children in recent time. However, the long-term trends in survival among adult leukemia patients and the associated sex and racial survival disparities are not well understood. We, therefore, evaluated the secular trends in survival improvement of leukemia patients from 1973 through 2014, using Surveillance Epidemiology and End-Result Survey Program (SEER) data. ICD-O-3 morphology codes were used to group leukemia into four types: acute lymphoblastic leukemia (ALL), acute myeloid leukemia (AML), chronic lymphocytic leukemia (CLL), chronic myeloid leukemia (CML). Survival analysis for each leukemia type stratified by race/ethnicity, age, sex was performed to generate relative survival probability estimates for the baseline time period of 1973 through 1979. Hazard ratios (HR) and respective 95% confidence intervals (CIs) for survival within subsequent 10-year time periods by race, age and sex were calculated using Cox proportional hazard models. Of the 83,255 leukemia patients for the current analysis, the 5-year survival of patients with ALL, AML, CLL, and CML during 1973–1979 were 42.0%, 6.5%, 66.5%, and 20.9%, respectively. Compared to the baseline, there were substantial improvements of leukemia-specific survival in 2010–2014 among African-American (81.0%) and Asian (80.0%) patients with CML and among 20–49 year of age with CLL (96.0%). African-American patients, those with AML and those older than 75 years of age had the lowest survival improvements. Asians experienced some of the largest survival improvements during the study period. Others, including African-American and the elderly, have not benefited as much from advances in leukemia treatment.

## Background

Leukemia is the 9^th^ most common cancer in the United States and the 6^th^ leading cause of cancer-related death with an incidence and mortality rates of 13.8 and 6.7 persons per 100,000 respectively [1]. Over the past several decades, despite advances in cancer diagnosis and treatment, leukemia incidence has continued to rise at 0.3% per year for the past decade with only an overall improvement in survival of about 1.5% annually [2].

Leukemia management is complicated by the fact that it is a heterogeneous group of diseases that affects both pediatric and adult populations, and demonstrates varying susceptibility to chemotherapeutic agents and prognosis [3]. For example, acute lymphoblastic leukemia (ALL) occurs predominantly in children and has a five-year survival of 90% [4], whereas in the early 2000s acute myeloid leukemia (AML), which affects predominantly the elderly, had a five-year survival rate of up to 15% in patients 60 years and older [5]. However, chronic leukemia which also affects adults tend to have a more favorable survival compared to AML, with the five-year survival of chronic myeloid leukemia (CML) and chronic lymphoid leukemia (CLL) reported as 69% and 66%, respectively, during 2007–2009 [6,7].

Although several factors are known to explain the survival differences in patients with leukemia, including access to more advanced therapy, strict inclusion criteria in clinical trials testing novel therapies which tends to exclude certain populations (i.e., minorities, the elderly and women), genetic differences (i.e., Philadelphia chromosome in CML), socioeconomic status and differences in patient preferences regarding treatment [8–12]; our knowledge of long term patterns of survival incorporating recent time periods among adults is limited. For instance, one of the more ethnically diverse studies among acute leukemia patients aged 15–75 years reported a five-year survival increase from 15.5% during 1991–1996 period to 22.5% during 2003–2008 period only [13]. Another study, spanning 1975–2012, which reported a five-year survival of 17.8% among AML patients in 2008–2012 from 12.7% in 1975–1981 was restricted to whites or African-Americans who were 39 years and younger [14]. Secular trends of leukemia survival remain fragmented by ethnicity/race and age. We, therefore, investigated the secular trends of survival among leukemia patients by ethnicity/race, age and sex from 1973 through 2014, using SEER data.

## Methods

The current analysis used data from the Surveillance Epidemiology and End Result (SEER) program, a population-based cancer data from 9 of its oldest registries for the time period 1973 through 2014. These registries cover approximately 10% of the U.S. population and are located in Atlanta, Georgia; Connecticut; Detroit, Michigan; Hawaii; Iowa; New Mexico; San Francisco-Oakland, California; Seattle–Puget Sound, Washington; and Utah [15]. The SEER registries in Connecticut, Hawaii, Iowa, New Mexico and Utah identified cancer cases from state-wide records, while those in Atlanta, California and Washington determined case eligibility in 5, 5 and 13 counties, respectively. Cancer cases that were reported to these registries were broadly defined as being diagnosed on or after January 1^st^ 1973, residents of the geographic area covered by SEER and having either an in-situ or invasive histologic tumor behavior, or a clinical diagnosis of malignancy [16].

The 3^rd^ Edition of the International Classification of Diseases for Oncology morphology codes (ICD-03) [16] was used to identify leukemia cases in the SEER dataset for the current analysis. We included patients who had an outcome of primary leukemia with known age and survival time. Leukemia records were abstracted using the following ICD-03 codes and classified as acute lymphoblastic leukemia (or ALL with the following codes: 9811, 9812, 9813, 9815, 9816, 9817, 9818, 9837), acute myeloid leukemia (or AML with the following codes: 9891, 9840, 9861, 9865, 9866, 9867, 9869, 9871–9874, 9895–9898, 9910, 9911, 9920), chronic lymphocytic leukemia (or CLL with the following code: 9823) and chronic myeloid leukemia (or CML with the following codes: 9863, 9875, 9876).

Other information that was obtained included age at diagnosis, race/ethnicity and marital status. Age at diagnosis was categorized into: 20–49, 50–64, 65–74 and 75 and older. The SEER race-ethnicity data comprised four original categories based on the 2000 United States Census and Bureau of Vital Statistics’ description of race, nationality and ethnicity: Non-Hispanic white, Non-Hispanic black, Hispanic and Non-Hispanic Asians [16]. Because the number of American Indian/Alaskan Native and Native Hawaiian/Pacific Island patients was small, these were included in and the Non-Hispanic Asian category were reclassified as “Other”. Per 2000 Census, patients who self-described as biracial, interracial mixed, multi-ethnic, national or racial were originally coded as race unknown but for the purpose of our analysis were incorporated into the race/ethnic “Other” category, except where they reported themselves as being of Hispanic ethnicity. Marital status was recoded such that small sized categories including divorced, widowed, and separated were grouped as “Other”. Persons with domestic partners were included with those who self-reported as “Married”.

The primary outcome was SEER-identified disease-specific mortality from leukemia. Cause of death was determined preferably by state department of health records or the National Death Index central database or state data exchanges [16]. Patient deaths which could only be confirmed by death certificate only were excluded from analysis. Survival time was defined from date of diagnosis to date last known to be alive, date of death or at study end on December 31, 2014. Patients who are still alive by study end or who had died of other causes during the study period were censored. SEER*STAT (version 8.3.2) was used to calculate 1-, 3- and 5- survival rates for each of the leukemia subtypes by race/ethnicity (i.,e., non-Hispanic whites, non-Hispanic blacks, Hispanics and Asian/Pacific Islanders), age (i.e., 20–49, 50–64, 65–74 and 75 years and older) and sex (i.e., male vs. female) using 1973–1979 period as a baseline (or reference) period.

We used Cox proportional hazards models to calculate Hazard Ratios (HRs) and their respective 95% confidence intervals (CIs) for leukemia-specific mortality among patients diagnosed during 1980 to 1989, 1990 to 1999, 2000 to 2009, and 2010 to 2014 periods and compared with those diagnosed at the baseline (1973 to 1979 period) for each race/ethnicity, age and sex group. We constructed approximate 10-year time periods to minimize small sample bias in our sub-group analysis. We assessed for interactions of the year of diagnosis with race/ethnicity, age and sex, respectively, for each of the four categories of leukemia using likelihood ratio tests [17,18], following similar strategy in our prior works [19,20], in which race/ethnicity, age or sex were included in multivariate models as covariates when they were not involved with interactions alongside marital status and cancer registry site.

Patient and leukemia-related characteristics were described by time periods, including the baseline, and presented in the supplementary tables (S1–S8 Tables). A Bonferroni correction was applied to the usual 0.05 level of significance to account for the increased probability of false positive results following 50 model specifications to obtain the final significant *P*-value of 0.001. All analyses were performed using SAS statistical software (version 9.4, SAS Institute Inc., Cary, NC).

## Results

During the 1973 through 2014 period, we identified 83,255 patients who were diagnosed with one of the major leukemia, including ALL (n = 4,807), AML (n = 27,559), CLL (n = 38,823), CML (n = 12,066). Men were more likely to be reported as leukemia cases than women, among all leukemia patients, the proportion of those with CLL steadily increased throughout the study period, from 43.2% in 1973–1979 to 49.4% in 2010–2014 (**Table 1 and S2–S6 Tables**).

**Table 1:** Distribution of leukemia cases by sex and year of diagnosis during 1973–2014 period in 9 SEER registries, (n = 83,255).

Cancer site				Year of diagnosis (n, %)									
		Total		1973–1979		1980–1989		1990–1999		2000–2009		2010–2014	
**ALL**	**Female**	2,025	(2.4)	169	(1.7)	405	(2.4)	472	(2.4)	589	(2.5)	390	(3.0)
	**Male**	2,782	(3.3)	262	(2.6)	592	(3.5)	688	(3.5)	817	(3.5)	423	(3.3)
**AML**	**Female**	12,589	(15.1)	1,591	(15.7)	2,459	(14.5)	3,021	(15.5)	3,607	(15.2)	1,911	(14.7)
	**Male**	14,970	(18.0)	1,842	(18.2)	3,069	(18.1)	3,658	(18.8)	4,227	(17.9)	2,174	(16.7)
**CLL**	**Female**	15,846	(19.0)	1,834	(18.1)	3,091	(18.2)	3651	(18.7)	4,721	(19.9)	2,549	(19.6)
	**Male**	22,977	(27.6)	2,536	(25.1)	4,558	(26.8)	5,138	(26.4)	6,866	(29.0)	3,879	(29.8)
**CML**	**Female**	5,202	(6.2)	816	(8.1)	1,249	(7.4)	1,232	(6.3)	1,178	(5.0)	727	(5.6)
	**Male**	6,864	(8.2)	1,063	(10.5)	1,558	(9.2)	1,619	(8.3)	1,667	(7.0)	957	(7.4)
**Total**		83,255	(100.0)	10,113	(100.0)	16,981	(100.0)	19,479	(100.0)	23,672	(100.0)	13,010	(100.0)

Abbreviations: ALL: Acute lymphoblastic leukemia AML: Acute myeloid leukemia CLL: Chronic lymphocytic leukemia CML: Chronic myeloid leukemia

Overall, there was an improvement in leukemia survival among all racial/ethnic groups, most age groups and between the sexes, with Asians/Pacific Islanders, patients 20–49 years of age and men demonstrating the largest improvement in 5-year relative survival (39.4%, 47.0% and 34.6%, respectively), from the 1973–1979 baseline period to the current 2010–2014 period (**Table 2**). Improvement in survival, on the other hand, was observed at a slower rate for patients 75 years and older at 24.4% during the same period. For patients diagnosed between 1973 and 1979, Hispanics had the lowest survival rates for ALL, Non-Hispanic whites for AML and ALL, and Asians for AML and ALL; those 75 years and older had the lowest rates across all leukemia types compared to other age groups; women had higher survival than men (**S1 Table**).

**Table 2:** Change of 5-year relative survival rate over time period of leukemia by age groups, race/ethnicity and sex in 9 SEER. Registries.

	1973–1979	1980–1989	1990–1999	2000–2009	2010–2014
**Age**	** **				
20–49	1,630 (28.4)	2,848 (35.1)	3,629 (48.8)	4,165 (64.8)	2,147 (75.4)
50–64	2,862 (43.6)	4,358 (48.9)	4,595 (55.4)	6,358 (68.4)	4,010 (74.3)
65–74	2,512 (37.5)	4,561 (43.0)	5,260 (48.2)	5,412 (60.3)	3,127 (65.7)
≥75	3,109 (26.7)	5,214 (31.8)	5,995 (34.9)	7,737 (45.4)	3,727 (51.1)
**Sex**					
Female	4,410 (38.1)	7,204 (43.7)	8,376 (50.8)	10,095 (61.3)	5,577 (67.1)
Male	5,703 (35.0)	9,777 (42.4)	11,103 (49.5)	13,577 (62.6)	7,433 (69.6)
**Race/ethnicity**					
Non-Hispanic White	8,960 (37.0)	14,542(44.3)	16,263 (51.4)	19,002 (63.2)	9,883 (69.7)
Non-Hispanic black	608 (32.4)	1,234 (35.4)	1,367 (42.4)	1,648 (54.5)	1,038 (61.6)
Hispanic (All Races)	262 (36.3)	533 (39.3)	808 (48.8)	1,327 (64.1)	927 (67.7)
Asian/Pacific Islander/(Other)	283 (27.0)	672 (35.4)	1,041 (43.5)	1,695 (56.3)	1,162 (66.4)

**Fig 1:**
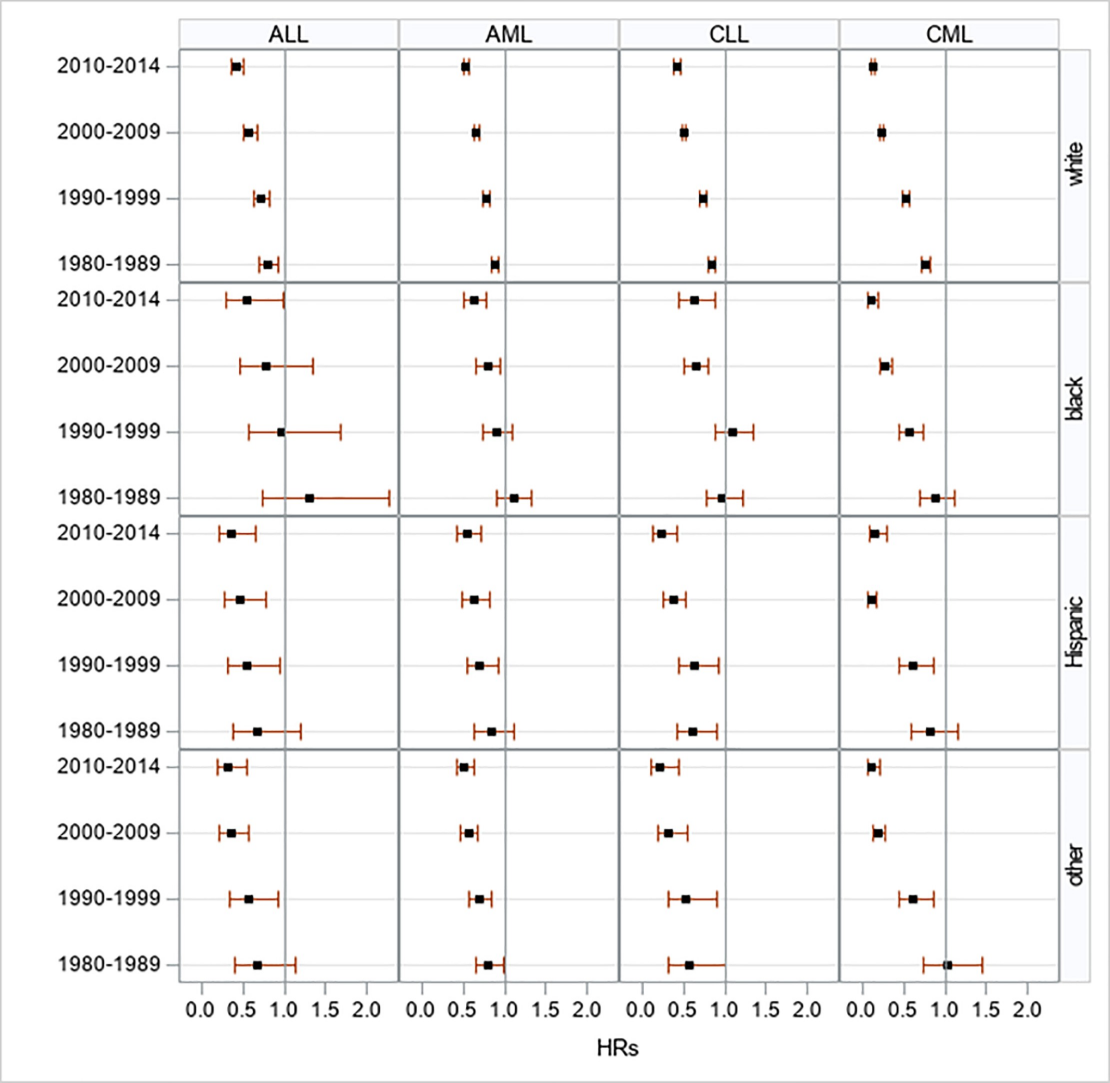
Multivariate-adjusted hazard ratios for cancer-specific death associated with year of diagnosis, according to race/ethnicity in 9 SEER registries, 1973–2014.

Although there have been improvements in survival, demonstrated by decreasing hazard ratios (HR) over time, for all types of leukemia during the study period among the four racial/ethnic groups, these trends reached significance among CML cases only (*P*<0.001). In particular, African-Americans and Asian patients with CML during 2009–2014 period experienced approximately 90% reductions in leukemia-specific mortality from the baseline period. African-Americans, otherwise, experienced the smallest increments in leukemia survival (**S6 Table and Fig 1**).

**Fig 2:**
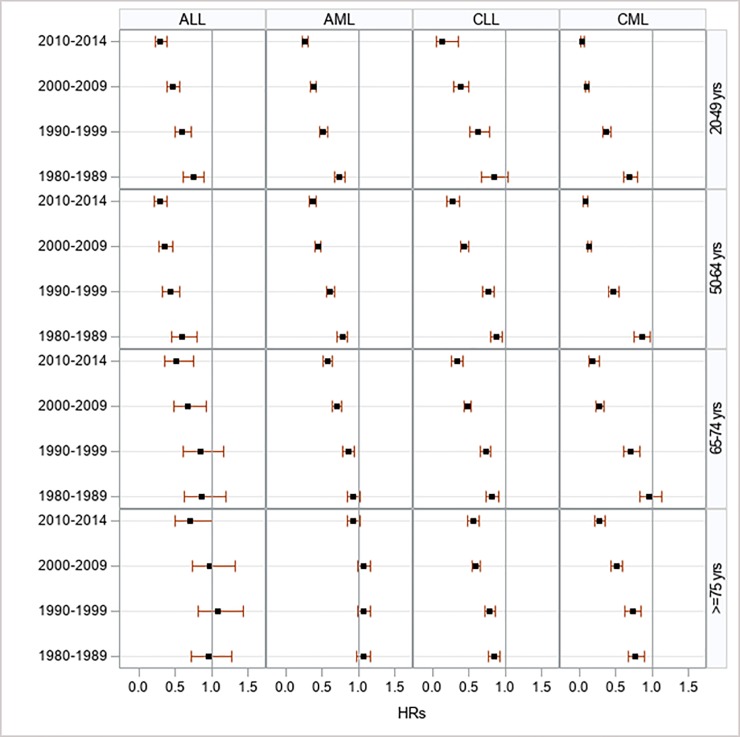
Multivariate-adjusted hazard ratios for cancer-specific death associated with year of diagnosis, according to age at diagnosis in 9 SEER registries, 1973–2014.

From 1973–2014, most age groups demonstrated improved survival across leukemia types. All interactions between the age groups and year of diagnosis were statistical significant (*P*<0.0001); being attributable to leukemia cases younger than 65 years of age, who demonstrated the largest survival gain during the study period. Accordingly, patients 20–49 years of age with CLL experienced a reduction of HR to 0.10 (95% CI: 0.03–0.28) in 2010–2014 from baseline. By contrast, the HRs and respective 95% CIs for those diagnosed with AML in the age group 75 years and older have remained unchanged since baseline and only declined afterwards (i.e., 2010–2014 period) to 0.91 (95% CI: 0.83–1.00) (**S7 Table and Fig 2**).

**Fig 3:**
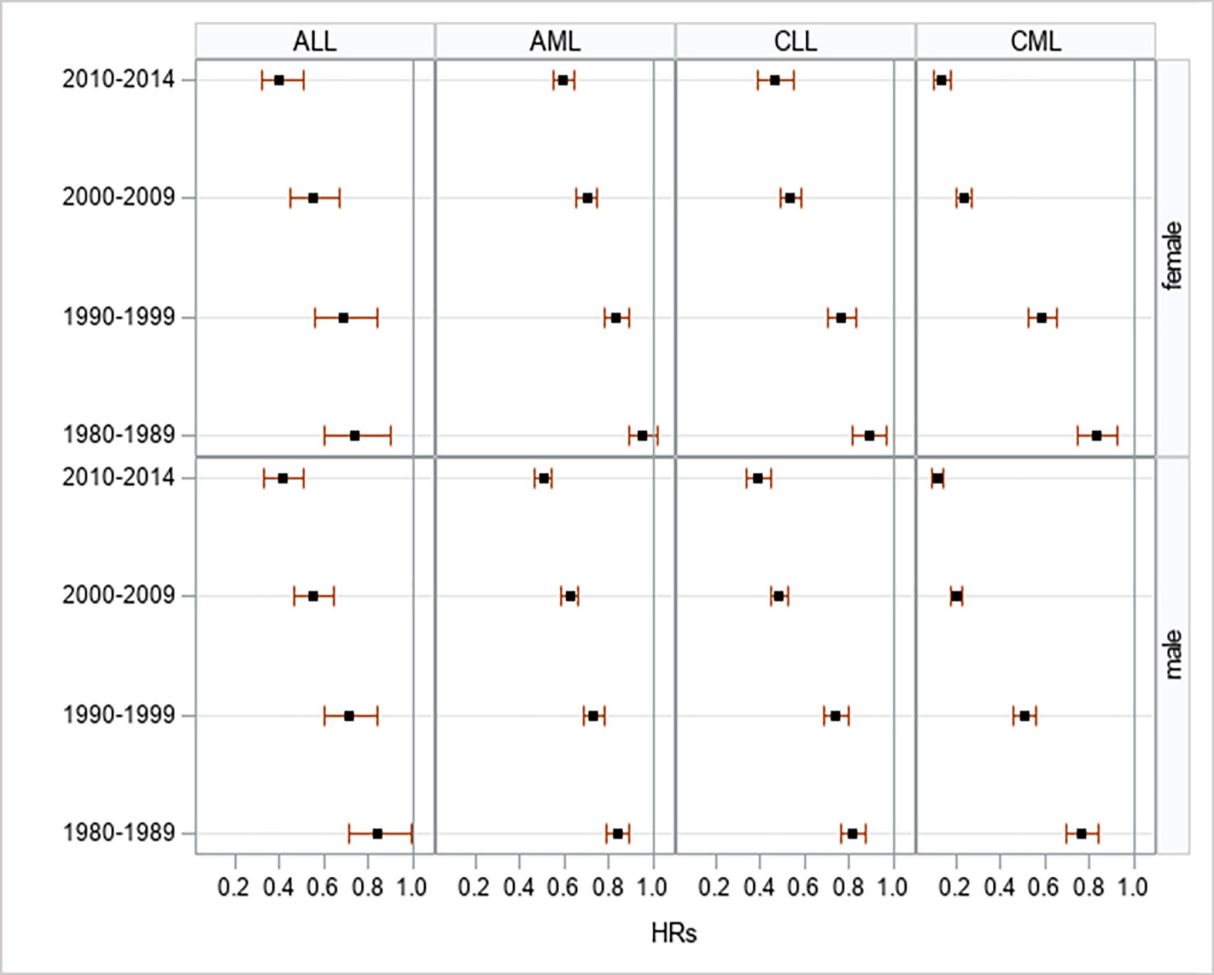
Multivariate-adjusted hazard ratios for cancer-specific death associated with year of diagnosis, according to sex at diagnosis in 9 SEER registries, 1973–2014.

Regarding the survival of leukemia by sex, we found that there was an improvement in cancer-specific survival for both men and women, most notably among ALL and CML cases. Men with CML experienced the most survival benefit with HR = 0.12 (95% CI: 0.09–0.17) in 2010–14. With the exception of ALL, women had poorer survival outcomes across virtually all time periods. However, these differences in survival were not reached to significant levels (**S8 Table and Fig 3**).

## Discussion

In this large nationally-representative sample of population-based cases of leukemia in the United States, we examined disparities during one of the most comprehensive time spans currently available. We observed an overall improvement in survival of leukemia patients across racial/ethnic, most age-groups and men and women during the period of 1973 through 2014. Although African-American patients and those older than 75 years of age experienced some of the smallest survival gains, Asians and persons younger than 50 years of age consistently displayed some of the largest decreases in leukemia-specific deaths. Consequently, there has been a widening survival disparity over time between Asians and African-Americans and persons at least 75 years of age and those less than 50, especially among AML patients.

Our findings should be interpreted with some cautions. The most recent time period is only 5 years long and results demonstrated therein should be considered preliminary and subject to small sample bias. SEER data exhibits variable reliability in coding rare cancer histology when compared to expert panel review. One study, for instance, reported an overall subtype agreement of 59% in a review of non-Hodgkin Lymphoma cases from the San Francisco Bay Area, despite the fact that it is one the SEER registries, positive predictive values among individual subtypes ranging from 5% to 100% [21]. At a minimum, we anticipate similar agreement with leukemia. Because the current study spans more than 40 years, it is especially susceptible to major revisions in diagnostic criteria. The World Health Organization (WHO) introduced and expanded the current ICD-O topographic and histologic format in its second and third iterations, respectively in 1990 and 2000 [16,22]. Much of the revisions to the definitions of hematologic malignancies adopted in these editions focused on better characterization of myeloproliferative diseases (MPD), myelodysplastic syndromes (MDS) and AML, with some old MDS entities assimilated into newer AML classifications. We, however, did not observe any increase in the proportion of AML cases between and beyond these time points to suggest any ensuing misclassification may have been substantial, nor do we have reason to believe a systematic bias affecting all nine participating registries occurred.

In the current analysis, we found that African-American with CML experienced the single largest reduction in leukemia-specific mortality among any racial/ethnic group. In fact, Asians experienced better survival improvements than any other racial/ethnic group across all leukemia subtypes. The reasons for this may be related to findings from CLL studies, which reveal earlier-than-average age at disease presentation, high socioeconomic status and high receipt of treatment among Asians [23,24]. Recent studies in equal access settings and our observation of drastic survival gains among African-American CML patients suggest that other factors such as the presence of poor karyotypes, higher treatment failures and complications may play additional roles in perpetuating survival disparities among minorities [25,26]. However, the widespread use of tyrosine kinase inhibitors (TKIs) in the 2000s, specifically imatinib, is credited for the subsequent and rapid improvement in CML survival [27]; prior to which, treatment involved either the use of stem cell transplantation (SCT) among a limited proportion of patients (30%) or interferon-α, with complete remission in up to 70% and 40% of these, respectively [28].

Our age-related findings were consistent with prior studies that demonstrated worsening survival among persons older than 65 years of age when compared to those that were younger [29–31]. This survival disparity was not only evident among all leukemia subtypes we examined but were also persistent across all time periods. In particular, among patients 75 years and older with AML, mortality has remained virtually unchanged; in part because of the presence of comorbidities, prior organ dysfunction, and a perception of being unfit for intensive chemotherapy [28]. As a result, for instance, only 33% of older patients with AML are offered any form of chemotherapy, with mortality rates as high as 50% within the first 6–8 weeks after the initiation of the standard of care [28]. On the other hand, improvements in AML survival among younger patients in the early 1980s and 2000s have been attributed to better understanding of prognostic cytogenic types, judicious use of SCT and better supportive care, such as less toxic conditioning agents, improvement in the treatment and prophylaxis of graft-versus-host disease and infection control [29]. Taken together, the evidence suggests that failure to initiate AML treatment among the elderly, without the attendant benefits of improved supportive care, may be the single most important reason for their continued poor survival.

We also found that leukemia-specific survival did not improve as fast for women as men, despite the former exhibiting better survival during the reference period. With the exception of ALL, men consistently but non-significantly exhibited a greater reduction in leukemia-specific mortality over time. These findings persisted after further evaluation of survival times among women and men. In the five most recent time periods, median and mean overall survival times were shorter among women than men, even though they were not statistically significant. Specifically, our sex-related finding among ALL patients was compatible with a study that reported favorable survival outcomes among females 19 years and younger with leukemia, when compared to age-adjusted males [32]. Although ALL accounts for the majority of leukemia cases in the pediatric population, we were unable to compare the rest of our findings with the this study [32] as their leukemia definition did not include chronic lymphoid or myeloid subtypes.

Our study has several strengths. To our knowledge, our study might be the first effort to determine the secular trends in leukemia patients by age, sex, race/ethnicity groups with the largest population to date, SEER is known to oversample foreign-born individuals, Hispanic, Asian and Native American populations, which is particularly useful in studying disparities. Furthermore, SEER data collection procedures remain a standard for population-based registries.

In conclusion, our study, to our knowledge, represents one of the most comprehensive assessments of the secular trends of leukemia survival disparity to date. It integrates results of often segregated major leukemia subtypes and racial/ethnic groups, with the aim of exploring the underlying contributors to survival disparities. Across race and ethnicities, most age groups and sex there has been marked improvement in leukemia survival over the past four decades. Asians experienced some of the largest survival improvements during the study period. However, long term progress in leukemia survival is hampered by sustained, excess mortality among African-Americans and the elderly.

## Supporting information

S1 Table Baseline relative survival rate (percentage) for leukemias by race/ethnicity, age and sex, 9 SEER cancer registries, 1973–1979 (n = 10,113). (DOCX)

S2 Table Acute lymphoblastic leukemia (ALL), 9 SEER cancer registries, 1973–2014. (DOCX)

S3 Table Acute myeloid leukemia (AML), 9 SEER cancer registries, 1973–2014. (DOCX)

S4 Table Chronic lymphocytic leukemia (CLL), 9 SEER cancer registries, 1973–2014. (DOCX)

S5 Table Chronic myeloid leukemia (CML), 9 SEER cancer registries, 1973–2014. (DOCX)

S6 Table Multivariable Hazard Ratios (HR) and 95% confidence intervals (CI) for interaction between year of diagnosis and race/ethnicity. (DOCX)

S7 Table Multivariable Hazard Ratios (HR) and 95% confidence intervals (CI) for interaction between year of diagnosis and age. (DOCX)

S8 Table Multivariable Hazard Ratios (HR) and 95% confidence intervals (CI) for interaction between year of diagnosis and sex. (DOCX)
