# Supplementary material for: Racial/ethnic, age and sex disparities in leukemia survival among adults in the United States during 1973-2014 period
Source: PLoS One. 2019 Aug 19;14(8):e0220864. doi: 10.1371/journal.pone.0220864 (PMC6699686; doi:10.1371/journal.pone.0220864)
Supplement: S1 Table — (DOCX) [file pone.0220864.s001.docx]

**S1 Table. Baseline Relative Survival Rate (Percentage) for Leukemias by Race/ethnicity,**

**Age and Sex, 9 SEER Cancer Registries, 1973-1979 (n=10,113)**

| **Survival Rate for** | **Overall** | **Race/Ethnicity** | | | | **Age** | | | | **Sex** | |
| --- | --- | --- | --- | --- | --- | --- | --- | --- | --- | --- | --- |
|  |  | **NHW** | **NHB** | **Hispanic** | **Asian**  **Pacific Islanders** | **20-49** | **50-64** | **65-74** | **≥75** | **Male** | **Female** |
| **ALL** |  |  |  |  |  |  |  |  |  |  |  |
| 1 year | 35.7 | 33.6 | 42.4 | 36.4 | 44.7 | 54.2 | 22.1 | 26.3 | 14.8 | 38.2 | 31.8 |
| 3 year | 15.5 | 13.0 | 17.1 | 6.1 | 23.6 | 22.3 | 6.7 | 14.1 | 9.6 | 15.8 | 14.8 |
| 5 year | 9.9 | 9.40 | 0.0 | 0.0 | 16.9 | 16.1 | 5.5 | 4.1 | 3.2 | 10.1 | 9.5 |
| **AML** |  |  |  |  |  |  |  |  |  |  |  |
| 1 year | 24.4 | 23.2 | 35.5 | 28.6 | 28.9 | 44.0 | 24.5 | 20.3 | 12.4 | 22.5 | 26.6 |
| 3 year | 7.9 | 6.8 | 10.1 | 10.5 | 11.0 | 16.4 | 6.9 | 5.3 | 4.3 | 6.5 | 9.5 |
| 5 year | 5.2 | 4.4 | 4.1 | 10.5 | 5.2 | 11.9 | 4.0 | 3.2 | 2.2 | 4.0 | 6.5 |
| **CLL** |  |  |  |  |  |  |  |  |  |  |  |
| 1 year | 87.4 | 87.9 | 84.9 | 81.9 | 72.7 | 96.2 | 94.6 | 90.0 | 77.3 | 85.9 | 89.6 |
| 3 year | 77.9 | 78.6 | 75.1 | 65.1 | 63.0 | 90.6 | 87.8 | 79.4 | 64.8 | 75.8 | 80.8 |
| 5 year | 67.0 | 68.9 | 60.4 | 59.7 | 49.9 | 80.4 | 77.8 | 68.0 | 52.7 | 64.4 | 70.6 |
| **CML** |  |  |  |  |  |  |  |  |  |  |  |
| 1 year | 66.4 | 64.5 | 75.4 | 81.8 | 80.0 | 85.2 | 76.5 | 61.3 | 43.0 | 65.2 | 67.9 |
| 3 year | 39.7 | 38.9 | 48.5 | 48.9 | 45.9 | 59.1 | 44.3 | 33.4 | 21.9 | 37.0 | 43.3 |
| 5 year | 21.0 | 21.5 | 12.2 | 20.1 | 25.1 | 30.2 | 24.9 | 18.9 | 9.6 | 18.7 | 23.9 |

ALL - acute lymphoblastic leukemia; AML - acute myeloid leukemia; CLL - chronic lymphocytic leukemia; CML - chronic myeloid leukemia

NHW - non-Hispanic whites; NHB - non-Hispanic blacks,

* includes lymphoid and myeloid subtypes not otherwise specified
