# Supplementary material for: Racial/ethnic, age and sex disparities in leukemia survival among adults in the United States during 1973-2014 period
Source: PLoS One. 2019 Aug 19;14(8):e0220864. doi: 10.1371/journal.pone.0220864 (PMC6699686; doi:10.1371/journal.pone.0220864)
Supplement: S2 Table — (DOCX) [file pone.0220864.s002.docx]

**S2 Table. Acute Lymphoblastic Leukemia (ALL), 9 SEER Cancer Registries, 1973-2014**

|  | | | | | | | | | | | | | | | | |
| --- | --- | --- | --- | --- | --- | --- | --- | --- | --- | --- | --- | --- | --- | --- | --- | --- |
|  | **Year of Diagnosis n (%)** | | | | | | | | | | | | | | | |
|  | **All** | | | **1973-1979** | | | **1980-1989** | | | **1990-1999** | | | **2000-2009** | | **2010-2014** | |
| **Age** | | | | | | | | | | | | | | | | |
| 20-49 | 2,367 | | (49.2) | 194 | (45.0) | 493 | | (49.4) | 587 | | (50.6) | 733 | | (52.1) | 360 | (44.3) |
| 50-64 | 1,141 | | (23.7) | 78 | (18.1) | 195 | | (19.6) | 250 | | (21.6) | 342 | | (24.3) | 276 | (33.9) |
| 65-74 | 621 | | (12.9) | 63 | (14.6) | 142 | | (14.2) | 151 | | (13.0) | 171 | | (12.2) | 94 | (11.6) |
| ≥75 | 678 | | (14.1) | 96 | (22.3) | 167 | | (16.8) | 172 | | (14.8) | 160 | | (11.4) | 83 | (10.2) |
| **Sex** | | | | | | | | | | | | | | | | |
| Female | 2,025 | | (42.1) | 169 | (39.2) | 405 | | (40.6) | 472 | | (40.7) | 589 | | (41.9) | 390 | (48.0) |
| Male | 2,782 | | (57.9) | 262 | (60.8) | 592 | | (59.4) | 688 | | (59.3) | 817 | | (58.1) | 423 | (52.0) |
| **Race/Ethnicity** | | | | | | | | | | | | | | | | |
| Hispanic (All Races) | | 572 | (11.9) | 20 | (4.6) | 71 | | (7.1) | 118 | | (10.2) | 209 | | (14.9) | 154 | (18.9) |
| Asian or Pacific Islander | | 481 | (10.0) | 27 | (6.3) | 72 | | (7.2) | 103 | | (8.9) | 187 | | (13.3) | 92 | (11.3) |
| Non-Hispanic Black | | 345 | (7.2) | 23 | (5.3) | 60 | | (6.0) | 79 | | (6.8) | 108 | | (7.7) | 75 | (9.2) |
| Non-Hispanic White | | 3,409 | (70.9) | 361 | (83.8) | 794 | | (79.6) | 860 | | (74.1) | 902 | | (64.2) | 492 | (60.5) |
| **Marital Status** | | | | | | | | | | | | | | | | |
| Married | 2,686 | | (55.9) | 247 | (57.3) | 589 | | (59.1) | 647 | | (55.8) | 758 | | (53.9) | 445 | (54.7) |
| Other | 935 | | (19.5) | 112 | (26.0) | 187 | | (18.8) | 243 | | (20.9) | 256 | | (18.2) | 137 | (16.9) |
| Single | 1,186 | | (24.7) | 72 | (16.7) | 221 | | (22.2) | 270 | | (23.3) | 392 | | (27.9) | 231 | (28.4) |
| **SEER Registry** | | | | | | | | | | | | | | | | |
| Atlanta | 432 | | (9.0) | 19 | (4.4) | 80 | | (8.0) | 104 | | (9.0) | 139 | | (9.9) | 90 | (11.1) |
| Connecticut | 685 | | (14.3) | 68 | (15.8) | 143 | | (14.3) | 175 | | (15.1) | 202 | | (14.4) | 97 | (11.9) |
| Detroit | 671 | | (14.0) | 57 | (13.2) | 136 | | (13.6) | 181 | | (15.6) | 186 | | (13.2) | 111 | (13.7) |
| Hawaii | 238 | | (5.0) | 17 | (3.9) | 50 | | (5.0) | 57 | | (4.9) | 77 | | (5.5) | 37 | (4.6) |
| Iowa | 617 | | (12.8) | 95 | (22.0) | 130 | | (13.0) | 142 | | (12.2) | 155 | | (11.0) | 95 | (11.7) |
| New Mexico | 368 | | (7.7) | 30 | (7.0) | 79 | | (7.9) | 81 | | (7.0) | 118 | | (8.4) | 60 | (7.4) |
| San Francisco | 810 | | (16.9) | 68 | (15.8) | 189 | | (19.0) | 184 | | (15.9) | 233 | | (16.6) | 136 | (16.7) |
| Seattle | 655 | | (13.6) | 50 | (11.6) | 115 | | (11.5) | 155 | | (13.4) | 208 | | (14.8) | 127 | (15.6) |
| Utah | 331 | | (6.9) | 27 | (6.3) | 75 | | (7.5) | 81 | | (7.0) | 88 | | (6.3) | 60 | (7.4) |
| **All** | 4,807 | | (100.0) | 431 | (100.0) | 997 | | (100.0) | 1,160 | | (100.0) | 1,406 | | (100.0) | 813 | (100.0) |
|  |  | |  |  |  |  | |  |  | |  |  | |  |  |  |
|  |  | |  |  |  |  | |  |  | |  |  | |  |  |  |
