# Supplementary material for: Racial/ethnic, age and sex disparities in leukemia survival among adults in the United States during 1973-2014 period
Source: PLoS One. 2019 Aug 19;14(8):e0220864. doi: 10.1371/journal.pone.0220864 (PMC6699686; doi:10.1371/journal.pone.0220864)
Supplement: S3 Table — (DOCX) [file pone.0220864.s003.docx]

| **S3 Table. Acute Myeloid Leukemia (AML), 9 SEER Cancer Registries, 1973-2014** | | | | | | | | | | | | |
| --- | --- | --- | --- | --- | --- | --- | --- | --- | --- | --- | --- | --- |
|  | **Year of Diagnosis n (%)** | | | | | | | | | | | |
|  | **ALL** |  | **1973-1979** | | **1980-1989** | | **1990-1999** | | **2000-2009** | | **2010-2014** | |
| **Age** |  |  |  |  |  |  |  |  |  |  |  |  |
| 20-49 | 6,094 | (22.1) | 757 | (22.1) | 1,251 | (22.6) | 1,510 | (22.6) | 1,729 | (22.1) | 847 | (20.7) |
| 50-64 | 6,500 | (23.6) | 904 | (26.3) | 1,265 | (22.9) | 1,405 | (21.0) | 1,853 | (23.7) | 1,073 | (26.3) |
| 65-74 | 6,462 | (23.4) | 781 | (22.7) | 1,381 | (25.0) | 1,729 | (25.9) | 1,674 | (21.4) | 897 | (22.0) |
| ≥75 | 8,503 | (30.9) | 991 | (28.9) | 1,631 | (29.5) | 2,035 | (30.5) | 2,578 | (32.9) | 1,268 | (31.0) |
| **Sex** | 12,589 | (45.7) | 1,591 | (46.3) | 2,459 | (44.5) | 3,021 | (45.2) | 3,607 | (46.0) | 1,911 | (46.8) |
| Female |  |  |  |  |  |  |  |  |  |  |  |  |
| Male | 14,970 | (54.3) | 1,842 | (53.7) | 3,069 | (55.5) | 3,658 | (54.8) | 4,227 | (54.0) | 2,174 | (53.2) |
| **Race/Ethnicity** | 1,482 | (5.4) | 91 | (2.7) | 199 | (3.6) | 321 | (4.8) | 503 | (6.4) | 368 | (9.0) |
| Hispanic (All Races) |  |  |  |  |  |  |  |  |  |  |  |  |
| Asian or Pacific Islander | 2,280 | (8.3) | 151 | (4.4) | 348 | (6.3) | 530 | (7.9) | 782 | (10.0) | 469 | (11.5) |
| Non-Hispanic Black | 1,990 | (7.2) | 196 | (5.7) | 407 | (7.4) | 456 | (6.8) | 591 | (7.5) | 340 | (8.3) |
| Non-Hispanic White | 21,807 | (79.1) | 2,995 | (87.2) | 4,574 | (82.7) | 5,372 | (80.4) | 5,958 | (76.1) | 2,908 | (71.2) |
| **Marital Status** | 16,337 | (59.3) | 2,165 | (63.1) | 3,387 | (61.3) | 4,043 | (60.5) | 4,510 | (57.6) | 2,232 | (54.6) |
| Married |  |  |  |  |  |  |  |  |  |  |  |  |
| Other | 7,763 | (28.2) | 939 | (27.4) | 1,569 | (28.4) | 1,827 | (27.4) | 2,254 | (28.8) | 1,174 | (28.7) |
| Single | 3,459 | (12.6) | 329 | (9.6) | 572 | (10.3) | 809 | (12.1) | 1,070 | (13.7) | 679 | (16.6) |
| **SEER Registry** | 2,085 | (7.6) | 185 | (5.4) | 380 | (6.9) | 508 | (7.6) | 649 | (8.3) | 363 | (8.9) |
| Atlanta |  |  |  |  |  |  |  |  |  |  |  |  |
| Connecticut | 4,120 | (14.9) | 613 | (17.9) | 865 | (15.6) | 960 | (14.4) | 1,100 | (14.0) | 582 | (14.2) |
| Detroit | 4,664 | (16.9) | 622 | (18.1) | 1,022 | (18.5) | 1,158 | (17.3) | 1,264 | (16.1) | 598 | (14.6) |
| Hawaii | 1,348 | (4.9) | 122 | (3.6) | 273 | (4.9) | 314 | (4.7) | 398 | (5.1) | 241 | (5.9) |
| Iowa | 3,901 | (14.2) | 594 | (17.3) | 761 | (13.8) | 972 | (14.6) | 1,094 | (14.0) | 480 | (11.8) |
| New Mexico | 1,553 | (5.6) | 150 | (4.4) | 249 | (4.5) | 361 | (5.4) | 488 | (6.2) | 305 | (7.5) |
| San Francisco | 4,325 | (15.7) | 629 | (18.3) | 972 | (17.6) | 1,026 | (15.4) | 1,107 | (14.1) | 591 | (14.5) |
| Seattle | 3,859 | (14.0) | 367 | (10.7) | 707 | (12.8) | 1,007 | (15.1) | 1,166 | (14.9) | 612 | (15.0) |
| Utah | 1,704 | (6.2) | 151 | (4.4) | 299 | (5.4) | 373 | (5.6) | 568 | (7.3) | 313 | (7.7) |
| **All** | 27,559 | (100.0) | 3,433 | (100.0) | 5,528 | (100.0) | 6,679 | (100.0) | 7,834 | (100.0) | 4,085 | (100.0) |
|  |  |  |  |  |  |  |  |  |  |  |  |  |
|  |  |  |  |  |  |  |  |  |  |  |  |  |
|  |  |  |  |  |  |  |  |  |  |  |  |  |
