# Supplementary material for: Racial/ethnic, age and sex disparities in leukemia survival among adults in the United States during 1973-2014 period
Source: PLoS One. 2019 Aug 19;14(8):e0220864. doi: 10.1371/journal.pone.0220864 (PMC6699686; doi:10.1371/journal.pone.0220864)
Supplement: S4 Table — (DOCX) [file pone.0220864.s004.docx]

| **S4 Table. Chronic Lymphocytic Leukemia (CLL), 9 SEER Cancer Registries, 1973-2014** | | | | | | | | | | | | | |
| --- | --- | --- | --- | --- | --- | --- | --- | --- | --- | --- | --- | --- | --- |
|  | **Year of Diagnosis n (%)** | | | | | | | | | | | | |
|  | **All** |  | | **1973-1979** | | **1980-1989** | | **1990-1999** | | **2000-2009** | | **2010-2014** | |
| **Age** | 2,312 | | (6.0) | 237 | (5.4) | 374 | (4.9) | 618 | (7.0) | 708 | (6.1) | 375 | (5.8) |
| 20-49 |  | |  |  |  |  |  |  |  |  |  |  |  |
| 50-64 | 11,378 | | (29.3) | 1,349 | (30.9) | 2,238 | (29.3) | 2,278 | (25.9) | 3,409 | (29.4) | 2,104 | (32.7) |
| 65-74 | 11,490 | | (29.6) | 1,279 | (29.3) | 2,420 | (31.6) | 2,793 | (31.8) | 3,140 | (27.1) | 1,858 | (28.9) |
| ≥75 | 13,643 | | (35.1) | 1,505 | (34.4) | 2,617 | (34.2) | 3,100 | (35.3) | 4,330 | (37.4) | 2,091 | (32.5) |
| **Sex** | 15,846 | | (40.8) | 1,834 | (42.0) | 3,091 | (40.4) | 3,651 | (41.5) | 4,721 | (40.7) | 2,549 | (39.7) |
| Female |  | |  |  |  |  |  |  |  |  |  |  |  |
| Male | 22,977 | | (59.2) | 2,536 | (58.0) | 4,558 | (59.6) | 5,138 | (58.5) | 6,866 | (59.3) | 3,879 | (60.3) |
| **Race/Ethnicity** | 1,069 | | (2.8) | 79 | (1.8) | 136 | (1.8) | 209 | (2.4) | 393 | (3.4) | 252 | (3.9) |
| Hispanic (All Races) |  | |  |  |  |  |  |  |  |  |  |  |  |
| Asian or Pacific Islander | 1,208 | | (3.1) | 33 | (0.8) | 115 | (1.5) | 201 | (2.3) | 455 | (3.9) | 404 | (6.3) |
| Non-Hispanic Black | 2,367 | | (6.1) | 247 | (5.7) | 486 | (6.4) | 543 | (6.2) | 665 | (5.7) | 426 | (6.6) |
| Non-Hispanic White | 34,179 | | (88.0) | 4,011 | (91.8) | 6,912 | (90.4) | 7,836 | (89.2) | 10,074 | (86.9) | 5,346 | (83.2) |
| **Marital Status** | 21,428 | | (55.2) | 2,555 | (58.5) | 4,453 | (58.2) | 4,944 | (56.3) | 6,129 | (52.9) | 3,347 | (52.1) |
| Married |  | |  |  |  |  |  |  |  |  |  |  |  |
| Other | 14,287 | | (36.8) | 1,570 | (35.9) | 2,701 | (35.3) | 3,133 | (35.6) | 4,471 | (38.6) | 2,412 | (37.5) |
| Single | 3,108 | | (8.0) | 245 | (5.6) | 495 | (6.5) | 712 | (8.1) | 987 | (8.5) | 669 | (10.4) |
| **SEER Registry** | 2,270 | | (5.8) | 179 | (4.1) | 384 | (5.0) | 432 | (4.9) | 768 | (6.6) | 507 | (7.9) |
| Atlanta |  | |  |  |  |  |  |  |  |  |  |  |  |
| Connecticut | 5,664 | | (14.6) | 687 | (15.7) | 1,146 | (15.0) | 1,274 | (14.5) | 1,631 | (14.1) | 926 | (14.4) |
| Detroit | 6,796 | | (17.5) | 887 | (20.3) | 1,542 | (20.2) | 1,628 | (18.5) | 1,813 | (15.6) | 926 | (14.4) |
| Hawaii | 836 | | (2.2) | 47 | (1.1) | 106 | (1.4) | 166 | (1.9) | 320 | (2.8) | 197 | (3.1) |
| Iowa | 7,140 | | (18.4) | 980 | (22.4) | 1,694 | (22.1) | 1,718 | (19.5) | 1,853 | (16.0) | 895 | (13.9) |
| New Mexico | 2,298 | | (5.9) | 201 | (4.6) | 332 | (4.3) | 541 | (6.2) | 826 | (7.1) | 398 | (6.2) |
| San Francisco | 5,244 | | (13.5) | 706 | (16.2) | 1,009 | (13.2) | 1,151 | (13.1) | 1,552 | (13.4) | 826 | (12.9) |
| Seattle | 6,369 | | (16.4) | 498 | (11.4) | 1,091 | (14.3) | 1,474 | (16.8) | 2,025 | (17.5) | 1,281 | (19.9) |
| Utah | 2,206 | | (5.7) | 185 | (4.2) | 345 | (4.5) | 405 | (4.6) | 799 | (6.9) | 472 | (7.3) |
| **All** | 38,823 | | (100.0) | 4,370 | (100.0) | 7,649 | (100.0) | 8,789 | (100.0) | 11,587 | (100.0) | 6,428 | (100.0) |
