# Supplementary material for: Racial/ethnic, age and sex disparities in leukemia survival among adults in the United States during 1973-2014 period
Source: PLoS One. 2019 Aug 19;14(8):e0220864. doi: 10.1371/journal.pone.0220864 (PMC6699686; doi:10.1371/journal.pone.0220864)
Supplement: S5 Table — (DOCX) [file pone.0220864.s005.docx]

| **S5 Table. Chronic Myeloid Leukemia (CML), 9 SEER Cancer Registries, 1973-2014** | | | | | | | | | | | | |
| --- | --- | --- | --- | --- | --- | --- | --- | --- | --- | --- | --- | --- |
|  | **Year of Diagnosis n (%)** | | | | | | | | | | | |
|  | **All** |  | **1973-1979** | | **1980-1989** | | **1990-1999** | | **2000-2009** | | **2010-2014** | |
| **Age** | 3,645 | (30.2) | 442 | (23.5) | 730 | (26.0) | 914 | (32.1) | 995 | (35.0) | 564 | (33.5) |
| 20-49 |  |  |  |  |  |  |  |  |  |  |  |  |
| 50-64 | 3,164 | (26.2) | 531 | (28.3) | 660 | (23.5) | 662 | (23.2) | 754 | (26.5) | 557 | (33.1) |
| 65-74 | 2,299 | (19.1) | 389 | (20.7) | 618 | (22.0) | 587 | (20.6) | 427 | (15.0) | 278 | (16.5) |
| ≥75 | 2,958 | (24.5) | 517 | (27.5) | 799 | (28.5) | 688 | (24.1) | 669 | (23.5) | 285 | (16.9) |
| **Sex** | 5,202 | (43.1) | 816 | (43.4) | 1,249 | (44.5) | 1,232 | (43.2) | 1,178 | (41.4) | 727 | (43.2) |
| Female |  |  |  |  |  |  |  |  |  |  |  |  |
| Male | 6,864 | (56.9) | 1,063 | (56.6) | 1,558 | (55.5) | 1,619 | (56.8) | 1,667 | (58.6) | 957 | (56.8) |
| **Race/Ethnicity** | 734 | (6.1) | 72 | (3.8) | 127 | (4.5) | 160 | (5.6) | 222 | (7.8) | 153 | (9.1) |
| Hispanic (All Races) |  |  |  |  |  |  |  |  |  |  |  |  |
| Asian or Pacific Islander | 884 | (7.3) | 72 | (3.8) | 137 | (4.9) | 207 | (7.3) | 271 | (9.5) | 197 | (11.7) |
| Non-Hispanic Black | 1,193 | (9.9) | 142 | (7.6) | 281 | (10.0) | 289 | (10.1) | 284 | (10.0) | 197 | (11.7) |
| Non-Hispanic White | 9,255 | (76.7) | 1,593 | (84.8) | 2,262 | (80.6) | 2,195 | (77.0) | 2,068 | (72.7) | 1,137 | (67.5) |
| **Marital Status** | 6,742 | (55.9) | 1,142 | (60.8) | 1,641 | (58.5) | 1,595 | (55.9) | 1,511 | (53.1) | 853 | (50.7) |
| Married |  |  |  |  |  |  |  |  |  |  |  |  |
| Other | 3,699 | (30.7) | 587 | (31.2) | 876 | (31.2) | 831 | (29.1) | 879 | (30.9) | 526 | (31.2) |
| Single | 1,625 | (13.5) | 150 | (8.0) | 290 | (10.3) | 425 | (14.9) | 455 | (16.0) | 305 | (18.1) |
| **SEER Registry** | 894 | (7.4) | 81 | (4.3) | 178 | (6.3) | 211 | (7.4) | 242 | (8.5) | 182 | (10.8) |
| Atlanta |  |  |  |  |  |  |  |  |  |  |  |  |
| Connecticut | 1,666 | (13.8) | 299 | (15.9) | 419 | (14.9) | 372 | (13.0) | 352 | (12.4) | 224 | (13.3) |
| Detroit | 2,349 | (19.5) | 428 | (22.8) | 587 | (20.9) | 555 | (19.5) | 509 | (17.9) | 270 | (16.0) |
| Hawaii | 466 | (3.9) | 60 | (3.2) | 99 | (3.5) | 112 | (3.9) | 126 | (4.4) | 69 | (4.1) |
| Iowa | 1,724 | (14.3) | 355 | (18.9) | 414 | (14.7) | 379 | (13.3) | 376 | (13.2) | 200 | (11.9) |
| New Mexico | 740 | (6.1) | 88 | (4.7) | 147 | (5.2) | 189 | (6.6) | 196 | (6.9) | 120 | (7.1) |
| San Francisco | 1,783 | (14.8) | 283 | (15.1) | 477 | (17.0) | 435 | (15.3) | 374 | (13.1) | 214 | (12.7) |
| Seattle | 1,701 | (14.1) | 193 | (10.3) | 338 | (12.0) | 412 | (14.5) | 476 | (16.7) | 282 | (16.7) |
| Utah | 743 | (6.2) | 92 | (4.9) | 148 | (5.3) | 186 | (6.5) | 194 | (6.8) | 123 | (7.3) |
| **All** | 12,066 | (100.0) | 1,879 | (100.0) | 2,807 | (100.0) | 2,851 | (100.0) | 2,845 | (100.0) | 1,684 | (100.0) |
