# Supplementary material for: Racial/ethnic, age and sex disparities in leukemia survival among adults in the United States during 1973-2014 period
Source: PLoS One. 2019 Aug 19;14(8):e0220864. doi: 10.1371/journal.pone.0220864 (PMC6699686; doi:10.1371/journal.pone.0220864)
Supplement: S6 Table — (DOCX) [file pone.0220864.s006.docx]

**S6 Table. Multivariable Hazard Ratios (HR) and 95% Confidence Intervals (CI) for**

**Interaction between Year of Diagnosis and Race/Ethnicity**

| **Race/ethnicity** | **1973-1979**  **(HR)** | **1980-1989**  **HR (95% CI)** | **1990-1999**  **HR (95% CI)** | **2000-2009**  **HR (95% CI)** | **2010-2014**  **HR (95% CI)** |
| --- | --- | --- | --- | --- | --- |
|  |  | **Acute Lymphoblastic Leukemia (ALL)** | | | |
| Non-Hispanic White | 1.00 | **0.79 (0.69-0.91)** | **0.71 (0.61-0.81)** | **0.56 (0.49-0.65)** | **0.41 (0.34-0.49)** |
| Non-Hispanic Black | 1.00 | 1.11 (0.65-1.89) | 0.98 (0.58-1.64) | 0.78 (0.47-1.30) | **0.50 (0.28-0.88)** |
| Hispanic | 1.00 | 0.75 (0.43 1.29) | **0.57 (0.34-0.97)** | **0.50 (0.30-0.83)** | **0.36 (0.21-0.63)** |
| Non-Hispanic Other | 1.00 | 0.68 (0.41-1.13) | **0.60 (0.37-0.98)** | **0.38 (0.24-0.61)** | **0.37 (0.21-0.63)** |
| *P_interaction_* | 0.771 |  |  |  |  |
|  |  | **­­­­Acute Myeloid Leukemia (AML)** | | | |
| Non-Hispanic White | 1.00 | **0.88 (0.84-0.92)** | **0.77 (0.74-0.81)** | **0.65 (0.62-0.39)** | **0.53 (0.50-0.57)** |
| Non-Hispanic Black | 1.00 | 1.09 (0.90-1.31) | 087 (0.72-1.05) | **0.78 (0.65-0.93)** | **0.61 (0.49-0.75)** |
| Hispanic | 1.00 | 0.86 (0.65-1.13) | **0.72 (0.55-0.93)** | **0.63 (0.49-0.82)** | **0.57 (0.43-0.74)** |
| Non-Hispanic Other | 1.00 | **0.80 (0.65-0.98)** | **0.69 (0.57-0.85)** | **0.55 (0.46-0.67)** | **0.51 (0.42-0.64)** |
| *P_interaction_* | 0.476 |  |  |  |  |
|  |  | **Chronic Lymphocytic Leukemia (CLL)** | | | |
| Non-Hispanic White | 1.00 | **0.84 (0.79-0.89)** | **0.73 (0.69-0.77)** | **0.50 (0.47-0.53)** | **0.41 (0.37-0.46)** |
| Non-Hispanic Black | 1.00 | 0.98 (0.79-1.21) | 1.08 (0.87-1.33) | **0.62 (0.50-0.77)** | **0.57 (0.40-0.80)** |
| Hispanic | 1.00 | **0.62 (0.43-0.91)** | **0.67 (0.47-0.95)** | **0.42 (0.30-0.60)** | **0.27 (0.14-0.50)** |
| Non-Hispanic Other | 1.00 | **0.56 (0.33-0.97)** | **0.47 (0.28-0.78)** | **0.28 (0.16-0.46)** | **0.22 (0.11-0.44)** |
| *P_interaction_* | 0.021 |  |  |  |  |
|  |  | **Chronic Myeloid Leukemia (CML)** | | | |
| Non-Hispanic White | 1.00 | **0.76 (0.70-0.82)** | **0.52 (0.48-0.56)** | **0.22 (0.20-0.24)** | **0.12 (0.10-0.15)** |
| Non-Hispanic Black | 1.00 | 0.88 (0.70-1.11) | **0.58 (0.46-0.74)** | **0.62 (0.21-0.36)** | **0.10 (0.05-0.18)** |
| Hispanic | 1.00 | 0.89 (0.64-1.23) | **0.64 (0.46-0.88)** | **0.11 (0.07-0.18)** | **0.15 (0.08-0.28)** |
| Non-Hispanic Other | 1.00 | 1.03 (0.73-1.43) | **0.60 (0.43-0.83)** | **0.18 (0.12-0.26)** | **0.10 (0.05-0.20)** |
| *P_interaction_* | <0.0001 |  |  |  |  |
