# Supplementary material for: Racial/ethnic, age and sex disparities in leukemia survival among adults in the United States during 1973-2014 period
Source: PLoS One. 2019 Aug 19;14(8):e0220864. doi: 10.1371/journal.pone.0220864 (PMC6699686; doi:10.1371/journal.pone.0220864)
Supplement: S7 Table — (DOCX) [file pone.0220864.s007.docx]

**S7 Table. Multivariable Hazard Ratios (HR) and 95% Confidence Intervals (CI) for Interaction between**

**Year of Diagnosis and Age**

| **Race/ethnicity** | **1973-1979**  **(HR)** | **1980-1989**  **HR (95% CI)** | **1990-1999**  **HR (95% CI)** | **2000-2009**  **HR (95% CI)** | **2010-2014**  **HR (95% CI)** |
| --- | --- | --- | --- | --- | --- |
|  |  | **Acute Lymphoblastic Leukemia (ALL)** | | | |
| 20-49 | 1.00 | **0.75 (0.62-0.91)** | **0.62 (0.51-0.74)** | **0.49 (0.40-0.59)** | **0.29 (0.22-0.38)** |
| 50-64 | 1.00 | **0.61 (0.46-0.81)** | **0.45 (0.34-0.59)** | **0.36 (0.27-0.47)** | **0.30 (0.22-0.40)** |
| 65-74 | 1.00 | 0.83 (0.60-1.14) | 0.83 (0.60-1.15) | **0.62 (0.45-0.85)** | **0.48 (0.33-0.70)** |
| ≥75 | 1.00 | 0.98 (0.74-1.30) | 1.18 (0.89-1.55) | 0.93 (0.70-1.24) | 0.74 (0.52-1.04) |
| *P_interaction_* | <0.0001 |  |  |  |  |
|  |  | **­­­­Acute Myeloid Leukemia (AML)** | | | |
| 20-49 | 1.00 | **0.73 (0.66-0.81)** | **0.51 (0.46-0.56)** | **0.37 (0.34-0.41)** | **0.26 (0.22-0.30)** |
| 50-64 | 1.00 | **0.78 (0.71-0.85)** | **0.61 (0.56-0.67)** | **0.45 (0.41-0.49)** | **0.37 (0.33-0.41)** |
| 65-74 | 1.00 | 0.92 (0.84-1.01) | **0.86 (0.79-0.94)** | **0.71 (0.64-0.77)** | **0.57 (0.50-0.63)** |
| ≥75 | 1.00 | 1.06 (0.97-1.15) | 1.06 (0.97-1.15) | 1.05 (0.97-1.10) | **0.91 (0.83-1.00)** |
| *P_interaction_* | <0.0001 |  |  |  |  |
|  |  | **Chronic Lymphocytic Leukemia (CLL)** | | | |
| 20-49 | 1.00 | 0.85 (0.69-1.05) | **0.65 (0.53-0.80)** | **0.39 (0.31-0.50)** | **0.10 (0.03-0.28)** |
| 50-64 | 1.00 | **0.87 (0.79-0.96)** | **0.77 (0.70-0.84)** | **0.42 (0.38-0.47)** | **0.21 (0.15-0.28)** |
| 65-74 | 1.00 | **0.82 (0.74-0.90)** | **0.73 (0.66-0.80)** | **0.47 (0.42-0.52)** | **0.30 (0.24-0.38)** |
| ≥75 | 1.00 | **0.83 (0.75-0.91)** | **0.77 (0.70-0.84)** | **0.59 (0.53-0.64)** | **0.61 (0.53-0.71)** |
| *P_interaction_* | <0.0001 |  |  |  |  |
|  |  | **Chronic Myeloid Leukemia (CML)** | | | |
| 20-49 | 1.00 | **0.67 (0.58-0.76)** | **0.34 (0.30-0.40)** | **0.10 (0.08-0.12)** | **0.03 (0.01-0.06)** |
| 50-64 | 1.00 | **0.86 (0.76-0.98)** | **0.50 (0.43-0.57)** | **0.13 (0.11-0.16)** | **0.07 (0.04-0.11)** |
| 65-74 | 1.00 | 0.95 (0.81-1.12) | **0.70 (0.60-0.83)** | **0.27 (0.22-0.33)** | **0.17 (0.12-0.26)** |
| ≥75 | 1.00 | **0.74 (0.65-0.85)** | **0.69 (0.60-0.80)** | **0.46 (0.39-0.53)** | **0.26 (0.20-0.34)** |
| *P_interaction_* | <0.0001 |  |  |  |  |
