# Supplementary material for: Racial/ethnic, age and sex disparities in leukemia survival among adults in the United States during 1973-2014 period
Source: PLoS One. 2019 Aug 19;14(8):e0220864. doi: 10.1371/journal.pone.0220864 (PMC6699686; doi:10.1371/journal.pone.0220864)
Supplement: S8 Table — (DOCX) [file pone.0220864.s008.docx]

**S8 Table. Multivariable Hazard Ratios (HR) and 95% Confidence Intervals (CI) for Interaction between Year of Diagnosis and Sex**

| **Race/ethnicity** | **1973-1979**  **(HR)** | **1980-1989**  **HR (95% CI)** | **1990-1999**  **HR (95% CI)** | **2000-2009**  **HR (95% CI)** | **2010-2014**  **HR (95% CI)** |
| --- | --- | --- | --- | --- | --- |
|  |  | **Acute Lymphoblastic Leukemia (ALL)** | | | |
| Male | 1.00 | **0.73 (0.59-0.89)** | **0.68 (0.56-0.83)** | **0.54 (0.44-0.66)** | **0.40 (0.32-0.50)** |
| Female | 1.00 | 0.85 (0.72-1.05) | **0.72 (0.61-0.85)** | **0.56 (0.48-0.66)** | **0.42 (0.34-0.51)** |
| *P_interaction_* | 0.975 |  |  |  |  |
|  |  | **­­­­Acute Myeloid Leukemia (AML)** | | | |
| Male | 1.00 | 0.95 (0.88-1.01) | **0.82 (0.77-0.88)** | **0.69 (0.64-0.73)** | **0.59 (0.54-0.64)** |
| Female | 1.00 | **0.84 (0.79-0.89)** | **0.73 (0.69-0.78)** | **0.62 (0.59-0.66)** | **0.50 (0.47-0.54)** |
| *P_interaction_* | 0.269 |  |  |  |  |
|  |  | **Chronic Lymphocytic Leukemia (CLL)** | | | |
| Male | 1.00 | **0.88 (0.81-0.96)** | **0.76 (0.69-0.82)** | **0.75 (0.48-0.58)** | **0.48 (0.40-0.57)** |
| Female | 1.00 | **0.81 (0.76-0.87)** | **0.74 (0.69-0.79)** | **0.48 (0.45-0.52)** | **0.38 (0.33-0.43)** |
| *P_interaction_* | 0.559 |  |  |  |  |
|  |  | **Chronic Myeloid Leukemia (CML)** | | | |
| Male | 1.00 | **0.82 (0.74-0.92)** | **0.59 (0.53-0.66)** | **0.24 (0.21-0.27)** | **0.12 (0.09-0.17)** |
| Female | 1.00 | **0.76 (0.69-0.83)** | **0.50 (0.45-0.55)** | **0.19 (0.17-0.22)** | **0.11 (0.09-0.15)** |
| *P_interaction_* | 0.552 |  |  |  |  |
